# Treatment outcomes based on radiation therapy fields for bifocal germinoma: Synchronous or disseminated disease?

**DOI:** 10.1371/journal.pone.0223481

**Published:** 2019-10-03

**Authors:** Seung Yeun Chung, Jung Woo Han, Dong-Seok Kim, Hong In Yoon, Chang-Ok Suh

**Affiliations:** 1 Department of Radiation Oncology, Yonsei Cancer Center, Yonsei University College of Medicine, Seoul, South Korea; 2 Department of Pediatrics, Severance Hospital, Yonsei University College of Medicine, Seoul, South Korea; 3 Department of Pediatric Neurosurgery, Severance Hospital, Yonsei University College of Medicine, Seoul, South Korea; 4 Department of Radiation Oncology, CHA Bundang Medical Center, CHA University, Seongnam-si, Gyeonggi-do, South Korea; Barretos Cancer Hospital, BRAZIL

## Abstract

Intracranial germinoma sometimes present as bifocal germinoma, and whether bifocal germinoma should be treated as a synchronous or disseminated disease remains unclear. This study aimed to determine the optimal treatment modality for bifocal germinoma. Patients with bifocal germinoma who received radiotherapy (RT) from March 1990 to August 2017 were included for analysis. A total of 21 patients were included. The median follow-up period was 76.2 months (range, 6.2–305.4 months). There were 17 patients who received cranio-spinal irradiation (CSI) with local RT; 3, whole ventricular RT (WVRT) with local RT; and 1, local RT only. Three recurrences occurred (1 patient each among those who underwent CSI, WVRT, and local RT). Recurrence in the patient who received CSI and who received WVRT occurred in the right thalamus and right frontal convexity, respectively. Meanwhile, the patient who received local RT showed not only a recurred lesion in the hypothalamus, but also cerebrospinal fluid seeding. For this patient, salvage CSI was performed and complete response was achieved after treatment. However, after 9 years and 6 months, he was diagnosed with glioblastoma and expired. As for toxicity, although 17 patients showed decrease in complete blood count levels during treatment, all patients recovered soon after treatment completion. Our findings suggest that bifocal germinoma may be considered as a disseminated disease when considering the patterns of failure according to RT fields. In addition, patients who received CSI showed low acute toxicity rates. However, further studies are necessary to confirm these findings.

## Introduction

Central nervous system (CNS) germ cell tumors are rare, accounting for only 0.5% of all primary brain and CNS tumors. Approximately 90% of these tumors develop in patients younger than 20 years [1]. Germinoma is a type of germ cell tumor that shows good prognosis after treatment. Intracranial germinomas present as bifocal germinoma in 6%-10% of patients, often synchronously in the pineal region and suprasellar area [1,2], and thus whether bifocal germinoma should be treated as a synchronous or disseminated disease is still debated. Some believe that bifocal germinoma is a synchronous local disease because of the excellent response to treatment, while others say it is a disseminated disease due to the subsequent leptomeningeal metastasis in some patients [3]. Currently, bifocal germinoma is primarily considered a disseminated disease in the United States, while it is considered a disseminated disease in Europe [3,4].

At present, the preferred radiation treatment (RT) field for patients with disseminated germinoma is cranio-spinal irradiation (CSI). However, although CSI has shown excellent results for non-disseminated germinoma, the current recommended RT technique for non-disseminated germinoma is reduced field RT such as whole brain RT or whole ventricular RT (WVRT) due to the toxicity of CSI [5–8]. Given that bifocal germinoma is yet to be established clearly as a synchronous or disseminated disease, the optimal RT field for bifocal germinoma also remains unclear.

This study aimed to determine whether bifocal germinoma is a synchronous or disseminated disease and identify the optimal treatment modality for such malignancy. Toward this goal, the treatment characteristics and outcomes of patients with bifocal germinoma were reviewed.

## Materials and methods

### Patients

**Fig 1:**
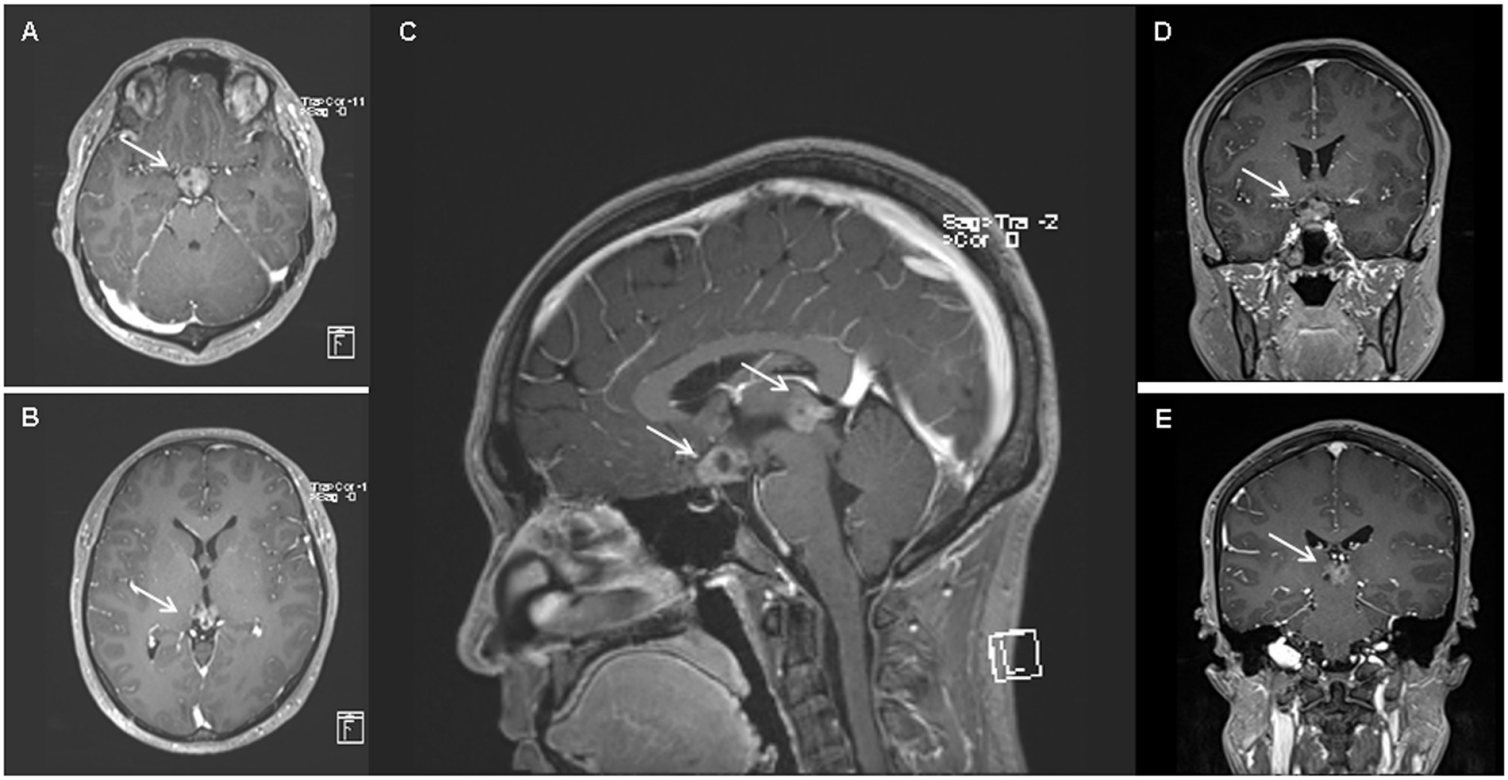
A case of bifocal germinoma at diagnosis. (a) Suprasellar lesion (white arrow) on MRI T1 gadolinium axial image. (b) Pineal lesion (white arrow) on MRI T1 gadolinium axial image. (c) Bifocal lesions (white arrows) on MRI T1 gadolinium sagittal image. (d) Suprasellar lesion (white arrow) on MRI T1 gadolinium coronal image; E) Pineal lesion (white arrow) on MRI T1 gadolinium coronal image.

We retrospectively reviewed the medical records of patients with germinoma who received RT between March 1990 and August 2017. Bifocal germinoma was defined as intracranial germinoma with another synchronous intracranial lesion (Fig 1). Patients with ventricular or spinal dissemination or lesions arising in the ventricles were excluded.

The study was approved by the Institutional Review Board (IRB No. 4-2018-1170). The informed consent was waived for this retrospective study.

### Radiotherapy fields

Three types of field were used for RT, namely, local RT, WVRT, and CSI, and these included the germinoma lesions, the whole ventricle, and the whole craniospinal axis, respectively. The RT field was decided upon the physician’s preferences.

### Treatment outcomes and statistical analysis

Patient, tumor, and treatment characteristics; treatment outcomes; and recurrence characteristics were retrospectively reviewed. Acute toxicity events were thoroughly reviewed retrospectively and graded following the National Cancer Institute Common Terminology Criteria for Adverse Events (version 4.03). Complete blood count levels during RT and after RT were also reviewed.

The cumulative probability of progression-free survival was calculated using the Kaplan-Meier method and compared using log rank test. All analyses were performed using SPSS version 23.0 (IBM Inc., Armonk, NY, USA).

## Results

### Patient characteristics

Of the 192 patients with germinoma who received RT, 21 patients with bifocal germinoma were included in the analysis. The median patient age was 18 years (range, 10–33 years), and most patients (86%) were male. The patient and tumor characteristics are shown in Table 1. Three of 4 patients who did not receive CSI had received prior chemotherapy, which included three regimens, namely, cisplatin/etoposide/bleomycin, cisplatin/etoposide, and carboplatin/etoposide/cyclophosphamide.

**Table 1:** Patient and tumor characteristics. Patient and tumor characteristics of 21 patients.

No	Sex	Location	Presenting symptom at diagmosis	Symptom duration (mo)	Endocrine dysfuction at diagnosis	Hydrocephalus	Pathologic diagnosis	Spine MRI	CSF cytology
1	F	Pineal gland, suprasellar	HA, blurred vision	36	DI	+	-	-	Negative
2	F	Pineal gland, suprasellar	Polydipsia, poor memory	1	DI	-	-	-	Negative
3	M	Pineal gland, suprasellar	HA	1		+	-	-	-
4	M	Pineal gland, pituitary stalk	HA, polydipsia, polyuria	3	DI	-	-	Negative	Negative
5	F	Thalamus, pituitary stalk	Lt. hemiparesis	12	Panhypotituitarism	-	Germinoma	-	-
6	M	Pineal gland, suprasellar	HA, N, V	1		+	-	-	-
7	M	Pineal gland, suprasellar	HA, polydipsia, polyuria	0.5	DI	-	-	-	-
8	M	Pineal gland, suprasellar	Polydipsia, polyuria	8	DI	-	Germinoma	-	-
9	M	Pineal gland, hypothalamus	HA, V, polyuria	1	DI	-	-	-	-
10	M	Pineal gland, pituitary stalk	Polydipsia, polyuria	3	DI	-	-	Negative	-
11	M	Basal ganglia, hypothalamus	HA, Lt. arm weakness	1		-	Germinoma	Negative	-
12	M	Pineal gland, pituitary stalk	Polydipsia, polyuria	4	DI	-	-	-	-
13	M	Pineal gland, hypothalamus	Polydipsia, polyuria	6	DI	-	Germinoma	Negative	-
14	M	Sellar, pineal gland	HA	1	DI	+	Germinoma	Negative	-
15	M	Hypothalamus, pineal gland	Polydipsia, polyuria, visual disturbance	3	DI	-	Germinoma	Negative	-
16	M	Pineal gland, suprasellar	HA	1	Panhypotituitarism	+	-	Negative	Negative
17	M	Pineal gland, suprasellar	HA, blurred vision	1	Panhypotituitarism	+	Germinoma	Negative	Negative
18	M	Pineal gland, suprasellar	Polydipsia, polyuria	5	DI	-	Germinoma	Negative	Negative
19	M	Pineal gland, suprasellar	Vision difficulty	4	Panhypotituitarism	+	Germinoma	Negative	Negative
20	M	Pineal gland, hypothalamus	HA, N, V, seizure	1		+	Germinoma	Negative	Negative
21	M	Pineal gland, hypothalamus	N, V, polydipsia, polyuria	1	DI	+	Germinoma	Negative	Negative

Abbreviations: F, female; M, male; HA, headache; N, nausea; V, vomiting; DI, diabetes insipidus; CSF, cerebrospinal fluid.

The most common tumor location was the pineal gland and suprasellar region. All patients had presenting symptoms at diagnosis such as headache and blurred vision, with a median symptom duration of 2 months (range, 1–36 months). A total of 17 patients (81%) showed endocrine dysfunction, and 9 patients (43%) had hydrocephalus. Approximately 50% of the patients (11 patients) received biopsy, and 1 patient underwent subtotal removal of the tumor. This patient showed tumor bleeding and left side weakness and thus underwent decompression surgery. One patient who underwent biopsy showed non-diagnostic results, and thus a total of 11 patients were pathologically confirmed with germinoma. The median alpha-fetoprotein (AFP) and total human chorionic gonadotropin (ThCG) at diagnosis were 2.39 ng/mL (range, 0.00–5.23 ng/mL) and 4.00 mIU/mL (range, 0.10–356.40 mIU/mL), respectively. Patients with elevated hCG were all pathologically diagnosed with germinoma. Patients who were difficult to perform biopsy due to tumor location were clinically diagnosed with neuroimaging studies and trial RT. Spinal magnetic resonance imaging (MRI) was performed in 12 patients (57%), and CSF cytology was checked in 9 patients (43%) to rule out seeding metastases.

### Analysis of treatment outcomes

The treatment characteristics are shown in Table 2. The largest RT field of each patient was local field RT only for 1 patient, WVRT for 3 patients, and CSI for 17 patients. Except 1 patient who received only local RT after systemic chemotherapy (patient No. 4), all patients were treated with local RT plus WVRT or CSI. The RT doses according to the RT field are as follows. For WVRT, a median total dose of 20 Gy (range, 18–20 Gy) with a fractional dose of 2 Gy; for whole-brain RT (WBRT), a median total dose of 18 Gy with a fractional dose of 1.8 Gy; for CSI, a median total dose of 19.5 Gy (range, 18–34.2 Gy) with a fractional dose of 1.5 or 1.8 Gy. The median local boost RT dose was 18 Gy (range, 7.5–21.6 Gy) with a median fractional dose of 1.8 Gy (range, 1.5–2.0 Gy). Six patients received trial RT. During RT, 8 patients (38%) had to rest with a median period of 4 days (range, 2–18 days) due to leukopenia (n = 3), MRI checking and replanning for trial RT (n = 4 patients), and equipment failure (n = 1).

**Table 2:** Treatment characteristics. Treatment characteristics including chemotherapy regimen and radiotherapy schedule.

No	Chemotherapy	Response after CTx			Recur
1	-		CSI	CSI 19.5 Gy+WB 18 Gy+Local 7.5 Gy	-
2	-		CSI	CSI 19.5 Gy+WB 18 Gy+Local 12 Gy	-
3	-		CSI	CSI 34.2 Gy+Local 20 Gy	-
4	DDP/Etoposide/Bleomycin #4	CR	Local field RT	Local 19.8 Gy	Yes
5	-		CSI	CSI 19.5 Gy+Local 19.8 Gy	Yes
6	-		CSI	CSI 19.5 Gy+Local 19.8 Gy	-
7	DDP/Etoposide #2	CR	CSI	CSI 19.5 Gy+Local 19.8 Gy	-
8	-		CSI	CSI 19.5 Gy+Local 19.8 Gy	-
9	DDP/Etoposide #4	PR	CSI	CSI 24.6 Gy+Local 16.2 Gy	-
10	-		CSI	CSI 19.5 Gy+Local 19.8 Gy	-
11	Carboplatin/Etoposide/Cyclophosphamide #6	PR	CSI	CSI 19.5 Gy+Local 19.8 Gy	-
12	-		CSI	CSI 24 Gy+Local 16.2 Gy	-
13	Carboplatin/Etoposide/Cyclophosphamide #6	CR	WVRT	WV 19.8 Gy+Local 10.8 Gy	-
14	-		CSI	CSI 24 Gy+Local 21.6 Gy	-
15	Carboplatin/Etoposide/Cyclophosphamide #4	PR	WVRT	WV 19.8 Gy+Local 10.8 Gy	-
16	-		CSI	CSI 19.5 Gy+Local 16 Gy	-
17	-		CSI	CSI 19.5 Gy+Local 16 Gy	-
18	-		CSI	CSI 19.5 Gy+Local 16 Gy	-
19	-		CSI	CSI 18 Gy+Local 18 Gy	-
20	-		WVRT	WV 18 Gy+Local 18 Gy	Yes
21	-		CSI	CSI 18 Gy+Local 18 Gy	-

Abbreviations: CTx, chemotherapy; CR, complete response; PR, partial response; CSI, cranio-spinal irradiation; RT, radiotherapy; WVRT, whole ventricle radiotherapy; WB, whole brain.

Chemotherapy was performed in 6 patients before RT. After chemotherapy, response evaluation before the start of RT showed 3 complete response (CR) and 3 partial response (PR). Out of the 3 patients who showed CR, 1 patient received local RT, 1 patient received WVRT, and the last patient received CSI. As for those who showed PR, 1 patient received WVRT and the other 2 patients received CSI.

During RT, 5 patients experienced grade 2 nausea. During RT, 3 and 1 patients developed grade 2 and 3 anemia, respectively. In addition, 6 and 11 patients experienced grade 2 and 3 white blood cell decrease, and 5 and 3 patients experienced grade 2 and 3 neutrophil count decrease, respectively. Grade 2 and 3 platelet count decrease was observed in 3 and 2 patients, respectively. Although many patients experienced hematologic toxicities during RT, the CBC levels of all patients were recovered soon after RT completion.

### Posttreatment recurrence

In total, 3 recurrences occurred after RT. The recurrence rates according to the RT field are shown in Table 3. Patients who received CSI showed the lowest recurrence rate, although the difference was not statistically significant (p = 0.08). Recurrence characteristics and salvage treatment of the patients who developed recurrence are shown in Table 4.

**Table 3:** Recurrences according to RT field. Recurrences according to cranio-spinal irradiation, whole ventricular radiotherapy and local field radiotherapy.

	Recur		
	No (n = 18)	Yes (n = 3)	
	n (%)	n (%)	p
CSI (n = 17)	16 (94.1)	1 (5.9)	0.08
WVRT (n = 3)	2 (66.7)	1 (33.3)	
Local field (n = 1)	0 (0.0)	1 (100.0)	

Abbreviations: CSI, cranio-spinal irradiation; WVRT, whole ventricular radiotherapy

**Table 4:** Recurrence characteristics and salvage treatment of patients with recurrence. Treatment and recurrence characteristics, salvage treatment and survival of patients with recurrence.

No	Chemotherapy	RT field	Recur at post-RT (mo)	Recur site	Salvage treatment		
4	DDP/Etoposide/Bleomycin #4	Local field RT	25.9	Hypothalamus, CSF seeding	Salvage CSI 27 Gy (brain 30Gy) + local 10.5 Gy	CR, but diagnosed with glioblastoma after 9Y6M	Dead
5	-	CSI	80	Rt. Thalamus	Follow up loss	-	Alive
20	-	WVRT	8.9	Rt. Frontal convexity	Tumor removal + on chemotherapy	-	Alive

Abbreviations: RT, radiotherapy; CSI, cranio-spinal irradiation; WVRT, whole ventricular radiotherapy; CSF, cerebrospinal fluid; Rt, right; CR, complete response

**Fig 2:**
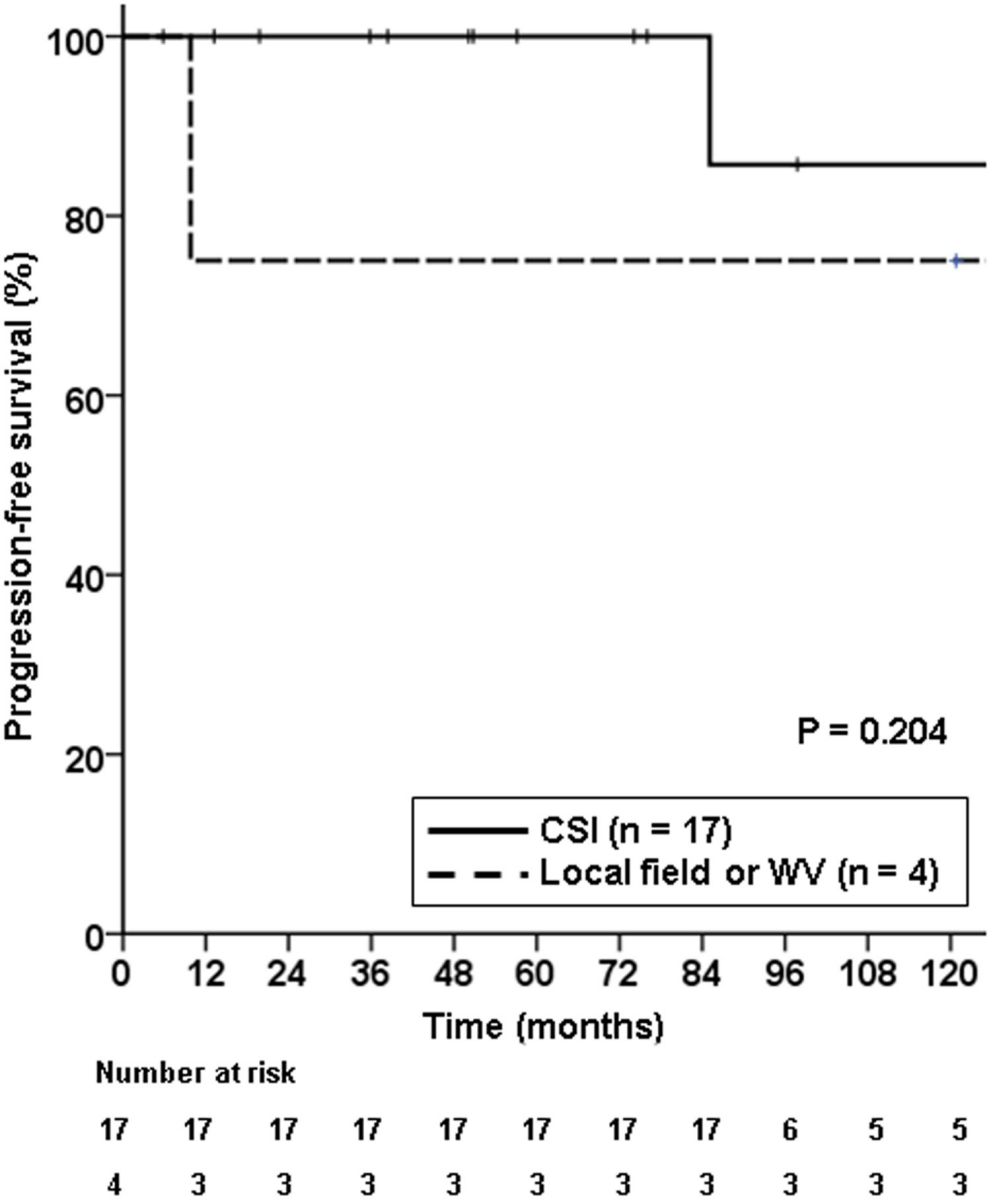
Progression-free survival according to the treatment field. Cranio-spinal irradiation tended to show better progression-free survival, although not statistically significant (p = 0.204)).

Recurrences occurred in those who received CSI, WVRT, and local field RT. The first recurrence case occurred in the CSI group (1/17). This patient initially showed bifocal germinoma at the thalamus and pituitary stalk, and the lesion was pathologically confirmed. She received CSI 19.5 Gy with local RT 19.8 Gy and developed recurrence 80 months after RT at the right thalamus. After recurrence, the patient moved to another hospital and is still alive. The second case occurred in the WVRT group (1/3 patients). The patient was pathologically diagnosed with bifocal germinoma at the pineal gland and hypothalamus and received WVRT 18 Gy and local RT 18 Gy without prior chemotherapy. At 8.9 months post-RT, recurrence occurred at the right frontal convexity, outside the RT field. On MRI, the recurred tumor had a possibility of both recurrence of germinoma and meningioma. Thus, gross total tumor resection was performed, and the lesion was pathologically proven as recurred germinoma. Currently, the patient is alive, is on chemotherapy, and is indicated for RT after the chemotherapy. The last patient who developed recurrence was the patient who received local RT only following systemic chemotherapy. He was diagnosed with bifocal germinoma at the pineal gland and pituitary stalk. Although this was not pathologically confirmed, the initial serum AFP was <5 ng/mL; ThCG, <5 mIU/mL; CSF AFP, 0.6 ng/mL; and beta hCG, <5 mIU/mL. Moreover, he did not show any evidence of seeding on both spinal MRI and CSF cytology. He received chemotherapy and showed CR prior to local RT 19.8 Gy. This patient showed recurrence at the hypothalamus and also CSF seeding at 25.9 months post-RT. He was treated with salvage CSI 27 Gy (brain: 30 Gy) + local RT 10.5 Gy. After salvage RT, the patient showed CR but was diagnosed with glioblastoma after 9 years and 6 months. This patient expired due to glioblastoma and was the only patient who expired out of the 21 patients. In addition, analysis of progression-free survival for the CSI group (n = 17) and local RT/WVRT group (n = 4) showed that the CSI group tended to show better progression-free survival than the local RT/WVRT group, although the difference was not statistically significant (p = 0.204; Fig 2).

## Discussion

Intracranial germinoma is known to be highly radiosensitive and has shown excellent treatment results. The standard treatment strategy for localized germinoma was a higher dose to the neuraxis and primary tumor using CSI and local RT [9]. However, long-term RT-induced toxicities were also observed although the survival rate improved to over 90%. Thereafter, many investigators have been interested in reduced RT volume and dose [5,10]. Recently, several studies have demonstrated that RT with reduced volume such as WVRT or WBRT showed excellent treatment outcome without severe long-term toxicity. Thus, reduced-volume RT is currently considered as the standard RT strategy for germinoma [11–13]. Meanwhile, CSI remains the standard RT field for disseminated germinoma. However, in cases of bifocal germinoma, there is no consensus on whether it should be treated as a synchronous disease or disseminated disease and thus, the optimal RT field remains unclear.

Since bifocal germinoma is rare, only cases reports and few retrospective studies exist [14–19]. Previous reports suggested that bifocal germinoma is a localized disease. Huang et al. reported 7 patients with bifocal germinoma who received WVRT of 30 Gy without chemotherapy without any recurrences [19]. Another retrospective study from Canada reported no recurrences in 6 patients with bifocal germinoma who received chemotherapy and WVRT [17]. However, some studies reported contradicting results. Ogawa et al. retrospectively studied 165 patients with no evidence of spinal metastases who were treated with cranial RT without spinal irradiation. Of these, 18 patients had bifocal tumor and 3 of the 18 patients had spinal recurrence after initial treatment [20]. In another retrospective study, out of 55 patients with bifocal germinoma, there were no failures in those who received CSI, 2 spinal failures in 17 patients who received limited RT field without chemotherapy, and 1 spinal failure in 23 patients who received limited RT field with chemotherapy [18]. In addition, another recent study from China reported no spinal recurrence in 23 patients treated with CSI, but 4 spinal failures in 24 patients treated with WVI or WBRT, thus advocating the use of CSI in bifocal germinoma patients [21].

In our study, the recurrence pattern of the patient who received local RT was not only intracranial recurrence, but also CSF dissemination. Meanwhile, the patient who recurred locally and who showed outfield intracranial recurrence received CSI and WVRT, respectively. Considering the pattern of failure in our study, bifocal germinoma seems to be a disseminating entity. Phi et al. have reported that of the 23 patients with bifocal germ cell tumor, 47.8% had tumor seeding at presentation, and tumor seeding was significantly associated with bifocal lesions (p < 0.001) [22]. Thus, they concluded that bifocal lesions may result from the metastatic spread of suprasellar or pineal lesions. Some researchers argued that local RT or WVRT with or without chemotherapy is feasible as definitive treatment due to the high CR rate and the effective salvage treatments after recurrence [17,19]. However, the patient who developed recurrence who received local RT in our institution was salvaged successfully via CSI with local RT. Thereafter, glioblastoma eventually developed, which may be considered a secondary malignancy because the sum of the irradiated dose to primary lesion was high (60.3 Gy) [23]. We consider that successful initial treatment is important because patients with bifocal germinoma tend to be young and have a long life expectancy and would be less exposed to cytotoxic treatment owing to successful initial treatment.

In addition, the location of the tumor may be a matter of concern for bifocal germinoma patients. Germinomas are most commonly located in the pineal region, followed by suprasellar region. Germinomas arising from basal ganglia and thalamus are rare and have atypical clinical presentation compared to those located in pineal or suprasellar region. They show variable radiologic findings, can cause atropic changes with neurologic deficits and the rate of treatment failure is reported to be higher [24–26]. In this study, the location of the tumor of the patient who recurred after local field RT was in the pineal gland and suprasellar area. As for the other two recurred cases, one case which was located in the thalamus and pituitary stalk recurred even with CSI, and the other case which was located in the hypothalamus and pineal gland recurred in the brain parenchyme after WVRT. Thus, local field RT may be insufficient for both bifocal germinomas located in the traditional areas and non-traditional areas.

As for toxicity, the acute toxicity rates were reasonable, and all patients recovered from hematologic toxicity after RT completion, showing that RT can be a tolerable treatment for bifocal germinoma patients. As for late toxicity, our study could not analyze neurocognitive function or quality of life due to its retrospective nature. However, there have been previous reports concerning this matter in patients with germinoma who received CSI. In germinoma patients, late toxicity after RT is generally negligible [13]. Merchant et al. reported no significant differences between pre- and post-RT full-scale, verbal, and performance IQ scores in 12 patients with germinoma who received CSI [27]. Another study reported a generally good quality of life in 22 adult survivors of germinomas treated with CSI [28]. Previous studies reporting poor neurocognitive outcome refer to CSI in other types of brain tumor, which shows different presentation compared to germinoma [29,30]. For example, patients with medulloblastoma show lower age at diagnosis, receives more aggressive surgery, and presents with more hydrocephalus, which increases the risk of neurocognitive damage [29,30]. Furthermore, the RT dose used for CSI in this study was mostly under 20 Gy, which is lower than in other tumor types such as medulloblastoma [31]. The risk and severity of chronic toxicity is increased with younger age and higher RT doses [32]. Asides from neurocognitive function, concerns for the development of bones and soft tissue for young patients, especially younger than the age of 10 years, can be raised [4]. Previous study reported that CSI administered at the age of 8 years or younger was related to short stature [33]. In this study, there were no patients younger than the age of 10 years. However, to treat younger bifocal patients, CSI by proton beam therapy can be considered to lower the radiation dose to the spine [34]. Furthermore, although one may suggest that CSI may be overtreatment and that it is low cost-effective, low dose CSI and omitting chemotherapy would be the most favorable option if CSI is regarded as a disseminated disease. Previously, the benefit of addition of chemotherapy was not demonstrated in disseminated germinoma patients [35]. Overall, CSI seems to be a reasonable treatment option for patients with bifocal germinoma in terms of both tumor control and toxicity.

There are limitations to this study due to its retrospective nature, including the lack of data for late toxicity. However, this study also has several strengths. First, the study included a relatively large number of patients with bifocal germinoma despite its single-center design. Moreover, acute toxicity was thoroughly reviewed and reported.

## Conclusions

Our findings suggest that bifocal germinoma may be considered as a disseminated disease when considering the patterns of failure according to RT fields. In addition, patients who received CSI showed low acute toxicity rates. However, further studies are necessary to confirm these findings.
